# Effect of Cr Content on the Microstructure and Toughness of the Supercritically Coarse-Grained Heat-Affected Zone in X80 Pipeline Steel

**DOI:** 10.3390/ma18153466

**Published:** 2025-07-24

**Authors:** Yuqin Qin, Feng Wang, Zhikui Li, Zhiguo Hu, Longyi Zhao, Shubiao Yin, Shujun Jia

**Affiliations:** 1 Faculty of Metallurgical and Energy Engineering, Kunming University of Science and Technology, Kunming 650093, China; 2 Engineering Steel Institute, Central Iron and Steel Research Institute Group, Beijing 100081, China; 3 PipeChina Engineering Technology Innovation Co., Ltd., Tianjin 300457, China; 4 Construction Project Management Branch of China National Petroleum Pipeline Network Group Co., Ltd., Langfang 065000, China

**Keywords:** X80 pipeline steel, Cr content, supercritically coarse-grained heat-affected zone, impact toughness

## Abstract

The existing studies mainly focus on the coarse-grained heat-affected zone and the inter-critically reheated coarse-grained heat-affected zone, while the studies on other sub-zones are relatively low. Meanwhile, the studies on the Cr element in steel mainly focus on the influence of the Cr element on strength and hardness; however, its mechanism is not very clear. Therefore, three kinds of X80 experimental steels with different Cr contents (0 wt.%, 0.13 wt.%, and 0.40 wt.%) were designed in this paper. The thermal simulation experiments on the supercritically coarse-grained heat-affected zone (SCCGHAZ) were carried out using a Gleeble-3500 thermal simulator. The effects of Cr on the microstructure and toughness of SCCGHAZ were systematically investigated through Charpy impact tests and microstructural characterization techniques. The results indicate that the microstructures of the three Cr-containing X80 experimental steels in SCCGHAZ are predominantly composed of fine granular bainite. However, impact tests at −10 °C show that the SCCGHAZs of 0 wt.% and 0.13 wt.% Cr steel exhibit higher impact energy, while that of the 0.40 wt.% Cr steel demonstrates significantly reduced energy impact (<100 J). Microstructural characterization reveals that the impact toughness of the SCCGHAZ in X80 steel is correlated with microstructural features, including effective grain size, grain boundary angles, and the volume fraction and shape of martensite–austenite (M-A) constituents. Among these factors, the volume fraction of M-A constituents substantially influences toughness. It was found that island-shaped M-A constituents inhibit crack propagation, whereas blocky M-A constituents impair toughness.

## 1. Introduction

The transition from coal in the Industrial Revolution to electricity in the 19th century, and then to oil and gas in the 20th century, highlights the central role of energy in driving social and economic progress. Even today, oil and gas remain essential resources [1,2]. Oil and gas pipelines, with their advantages of safety, efficiency, and environmental friendliness, dominate the field of hydrocarbon transportation and are responsible for delivering over 90% of the world’s oil and gas. However, long-distance oil and gas pipeline projects involve large-scale forming welds and installation welds. Welding is a specialized heat treatment process in which the joint microstructure undergoes rapid heating and cooling, leading to significant microstructural changes that directly affect the safety of pipeline operation [3,4,5]. Among these, field girth welds are more challenging than longitudinal welds, as girth welding typically involves multi-pass and multi-layer welding, resulting in a more complex secondary HAZ. Therefore, to ensure that the pipeline steel goes through “high-temperature non-uniform rapid heating-short-term insulation-rapid cooling” after the welding thermal cycle of the HAZ while maintaining organizational uniformity and excellent performance, ensuring the safe and reliable service of oil and gas pipelines is one of the key factors. The coarse-grained heat-affected zone (CGHAZ) and the inter-critically reheated coarse-grained heat-affected zone (ICCGHAZ) are typically considered the regions with the poorest toughness in welded joints, as they tend to form coarse austenite grains and detrimental microstructures (e.g., chain-like martensite–austenite constituents) during the welding process. Consequently, they have become a major focus of current research, both domestically and internationally. In contrast, the supercritically coarse-grained heat-affected zone (SCCGHAZ) undergoes two-phase transformation and recrystallization processes during the welding thermal cycle, with its secondary peak temperature ranging between Ac_3_ and ~1100 °C. Within this temperature range, the newly formed austenite grains have limited time to grow, resulting in a significantly finer microstructure [6]. The SCCGHAZ generally exhibits superior low-temperature toughness compared to the CGHAZ and ICCGHAZ. Accordingly, research on the SCCGHAZ has been relatively limited.

In the HAZ, the study of martensite–austenite (M-A) constituents and the high-angle grain boundary (HAGB) is necessary to unravel the SCCGHAZ toughness mechanism. There are three main morphologies of M-A constituents: massive, elongated, and island [7]. Nellikode et al. [8] found that the M-A constituents of the ICCGHAZ are coarse in size and distributed in blocks and chains, which determine crack nucleation and extension. Li [9] and Di et al. [10] suggested that long, striped M-A constituents with lower interfacial energies are more prone to debonding from the matrix, while blocky M-A constituents are more prone to rupture. Lan et al. [7] found that thick and long striped M-A constituents contribute to microcrack initiation, which was further verified using Griffith’s classical theory, and found that the critical stress$,\;\sigma_{c}$, becomes smaller when the average width of the M-A constituent becomes larger, and thus, crack nucleation occurs when the centralized localized stress in the M-A constituent exceeds $\sigma_{c}$, which may lead to crack extension across the M-A constituent/matrix interface. Luo et al. [11] found that island-shaped M-A constituents are harmless to toughness, elongated M-A constituents are composed of almost all martensite, which has the lowest toughness, while blocky M-A constituents have a core–shell structure, with martensite as the “shell” and austenite as the “core”, which contributes more to the toughness than elongated M-A constituents. Additionally, with the increase in both the content and size of M-A constituents, the ductile-to-brittle transition temperature rises markedly, resulting in a significant deterioration of material toughness [12]. When the content of M-A constituents in the material exceeds 6%, the effect of M-A constituents on impact toughness significantly exceeds the proportion of HAGBs [13]. HAGBs effectively hinder or even prevent the linear propagation of cleavage cracks, thereby significantly enhancing impact toughness [14,15,16]. The higher the proportion of the HAGB, the more energy is consumed during crack propagation. Meanwhile, the HAGB also serves as an important indicator for evaluating EGS [17].

Microalloying elements are an important factor in controlling the microstructure and mechanical properties of the heat-affected zone [18,19,20]. Research on Cr in high-strength, low-alloy steels has shown that this element is beneficial to both strength and hardness [21,22,23,24,25]. Wu et al. [26] found that by reducing the amount of Mo alloying and adding a small amount of Cr alloying in thick-walled pipeline steel, the microstructure of the pipeline steel still primarily consisted of ferrite + bainite. Moreover, the formation of M-A constituents could be effectively avoided, thereby enhancing the low-temperature impact toughness of the pipeline steel. However, Yao et al. [27] found that the addition of Ni and Cr did not significantly improve the cryogenic impact toughness of low-carbon bainitic/martensitic multiphase steel. There is limited research on the effects of Cr addition on the microstructure and toughness of X80 pipeline steel, particularly on the HAZ. Therefore, three X80 experimental steels with different Cr contents were prepared in this study. The SCCGHAZ was simulated using a Gleeble-3500 thermal simulator (Poestenkill, NY, USA) under conditions where only the peak temperature of the secondary thermal cycle varied. Their low-temperature toughness and microhardness were analyzed, while the microstructure of thermal simulation specimens with a peak temperature of 1000 °C was characterized in detail through OM, SEM, and TEM to elucidate the relationship between Cr content and microstructural features. Combined with fracture analysis, the mechanical properties of the materials were comprehensively evaluated, thereby providing safety assurance for oil and gas transportation.

## 2. Experimental Procedures

### 2.1. Experimental Materials

**Figure 1:**
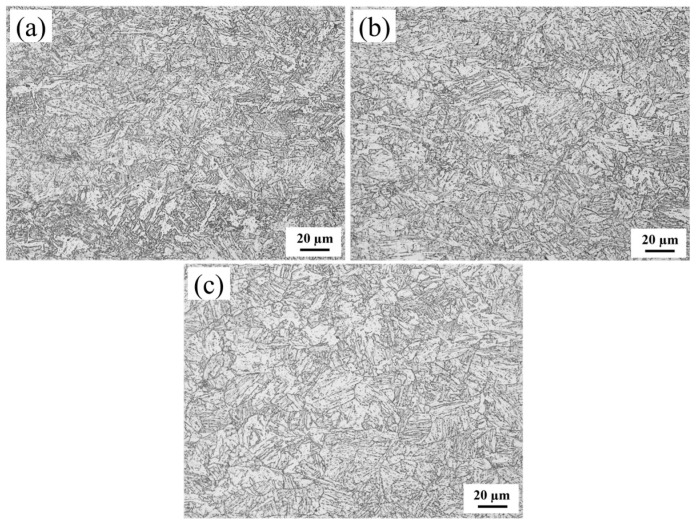
OM of specimens. (**a**) 0Cr; (**b**) 0.1Cr; (**c**) 0.4Cr.

To investigate the influence of the Cr content on the toughness of the HAZ in fully automated welded joints of X80 pipeline steel, this study utilized laboratory-melted and rolled X80 experimental steel in a thermomechanically controlled processing (TMCP) state. Chemical composition analysis was conducted using a SPECTROLAB metal analyzer (Kleve, Germany), with key components listed in Table 1. Based on Cr content variations, the three experimental steels were designated as 0Cr, 0.1Cr, and 0.4Cr. The matrix microstructure of 0Cr steel and 0.1Cr steel is predominantly composed of granular bainite (GB), as shown in Figure 1a,b. GB is a product of medium-temperature transformation and is formed through a mixed mechanism of shear and diffusion. Under OM and SEM, GB typically appears as irregular blocky structures. However, it is actually composed of laths, whose morphology can be resolved using TEM. In contrast, the matrix microstructure of 0.4Cr steel is primarily composed of bainitic ferrite (BF). Both BF and GB are medium-temperature transformation products that share the same transformation mechanism. However, BF forms at a lower transformation temperature, resulting in a stronger tendency toward lath formation. As shown in Figure 1c, BF laths grow from the prior austenite grain boundaries into the grain interior in a parallel manner. Different sets of BF laths with varying orientations divide the original austenite grains into distinct regions.

### 2.2. Welding Thermal Simulation Procedures

**Figure 2:**
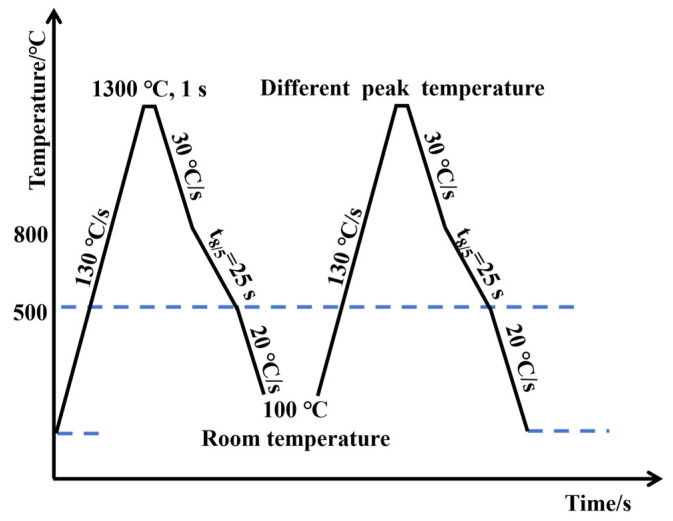
Schematic diagram of the thermal cycling process simulating different secondary peak temperatures.

Specimens with dimensions of 10 mm × 10 mm × 70 mm were machined from the mid-thickness region of the steel plate along the rolling direction. The microstructure of the simulated SCCGHAZ was reproduced using a Gleeble-3500 thermal simulator. The thermal cycle parameters (see Figure 2 for details) were as follows: specimens were heated uniformly at a rate of 130 °C/s to 1300 °C, held for 1 s, and then cooled at a specified rate to 100 °C, with a t8/5 cooling time set to 25 s. Secondary thermal cycles were conducted with peak temperatures of 900 °C, 950 °C, 1000 °C, and 1100 °C, and four specimens were prepared for each peak temperature.

### 2.3. Microstructural Characterization and Mechanical Properties Test

**Figure 3:**
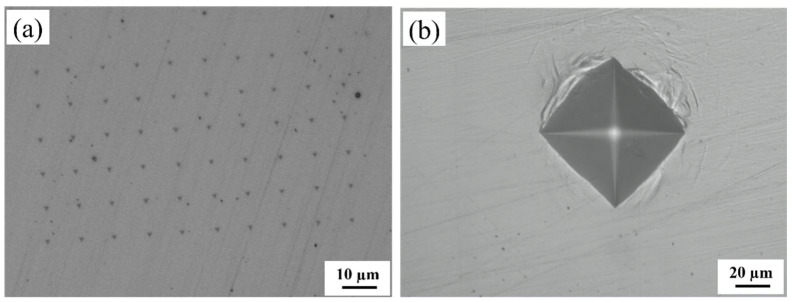
Indentation morphology of the experimental steel: (**a**) nanoindentation morphology; (**b**) Vickers hardness indentation morphology.

Following thermal cycling, the impact specimens were machined and notched (10 mm × 10 mm × 55 mm, with a notch depth of 2 mm) in accordance with the national standard, GB/T 229-2020 [28], and tested at −10 °C, with three parallel specimens specified for each set of experiments. In addition, thermal simulation specimens with dimensions of 10 mm × 10 mm × 10 mm were machined for microscopic characterization. The hardness of the surface of the thermal simulation specimens was determined using an MH-500D semi-automatic micro-Vickers hardness tester (Shanghai, China) with a load setting of 500 g and five points for each thermal simulation specimen. An Agilent G200 nanoindentation tester (Santa Clara, CA, USA) was used for hardness testing of the M-A constituents in thermally simulated specimens. The hardness of the M-A constituent in the thermal simulation specimen was measured using an Agilent G200 nanoindentation system. The test was conducted in displacement-controlled mode with the following parameters: a constant loading strain rate of 0.05 s^−1^, a maximum indentation depth of 200 nm, and a Poisson’s ratio of 0.25. A 6 × 10 matrix array was arranged on the sample surface, with a spacing of 10 µm between adjacent points to ensure the independence of each indentation, as shown in Figure 3a [29].

The microstructure of the thermally simulated specimen was characterized using an Olympus GX 53 optical microscope (OM) (Tokyo, Japan) and a thermal field-emission FEI Quanta 650 FEG scanning electron microscope (SEM) (Hillsboro, OR, USA). Prior to observation, the cross-section was mechanically ground stepwise (150# to 1000#), followed by mechanical polishing to ensure a flat and defect-free surface. The sample was then etched using a 4% nitric acid–96% ethanol solution. Electron backscatter diffraction (EBSD) was employed to acquire crystal orientation information, and the data were processed using HKL-Channel 5 software. After color etching of the specimen surface using Lepera reagent, Image-Pro Plus software (https://mediacy.com/image-pro/) was used to statistically analyze M-A constituents with different morphologies in 200× magnification fields. A Bruker D8ADVANCE X-ray diffractometer (XRD) (Billerica, MA, USA) was utilized to obtain key parameters, such as peak positions, intensities, and full width at half maximum (FWHM). The target material was Co target, with a tube current of 40 mA, a tube voltage of 35 kV, and a scanning speed of 2°/min.

## 3. Results

### 3.1. Mechanical Property

**Figure 4:**
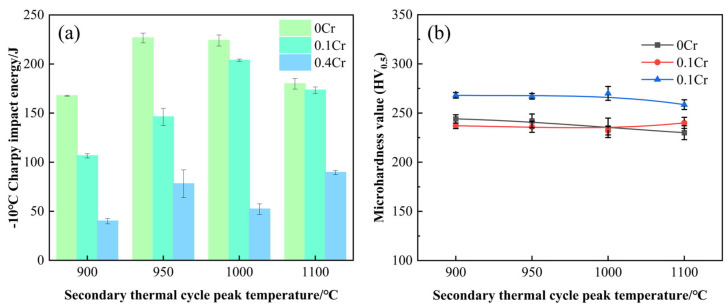
Mechanical properties of experimental steels with different Cr contents in the SCCGHAZ: (**a**) impact energy; (**b**) microhardness.

Figure 4 illustrates the relationship between the microhardness and the impact toughness of experimental steels with different Cr contents and the peak temperature of the secondary thermal cycle. As seen in Figure 4a, with increasing peak temperature of the secondary thermal cycle, the impact energy of 0Cr steel and 0.1Cr steel first increases and then decreases, while that of 0.4Cr steel fluctuates below 100 J. At the same peak temperature, the impact energy varies significantly among steels with different Cr contents. Among them, 0Cr steel exhibits the highest impact energy, followed by 0.1Cr steel, while 0.4Cr steel shows the lowest values. Figure 4b presents the microhardness of the SCCGHAZ in the experimental steels. The results indicate that the microhardness of the three steels remains relatively stable across different peak temperatures. Specifically, 0Cr steel and 0.1Cr steel exhibit microhardness values around 240 HV_0.5_, whereas 0.4Cr steel shows higher values near 270 HV_0.5_. According to Ref. [30], the secondary thermal cycle promotes recrystallization and microstructural homogenization in the SCCGHAZ, leading to stabilized microstructures and thus maintaining hardness at a consistent level. However, under identical peak temperature conditions, the microhardness gradually increases with higher Cr content.

### 3.2. Microstructure Characterization

**Figure 5:**
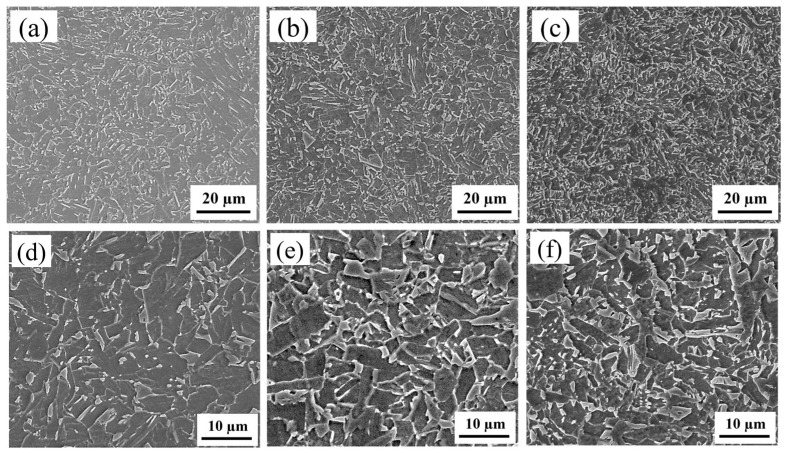
OM and SEM images of X80 experimental steels with different Cr contents (1000 °C): (**a**,**d**) 0Cr steel; (**b**,**e**) 0.1Cr steel; (**c**,**f**) 0.4Cr steel.

The SCCGHAZ is fully austenitized after secondary thermal cycling, but because the peak temperature is lower than the rapid grain growth temperature, a fine microstructure eventually forms. Fine grains promote more uniform dispersion of deformation under external forces, reducing stress concentration and strain differences between grain interiors and boundaries, thereby inhibiting crack initiation. Even if microcracks form, the increased grain boundaries hinder their propagation and dissipate more energy, thereby granting the SCCGHAZ superior impact toughness [31]. However, as can be seen in Figure 4a, the impact energy of 0.4Cr steel at the SCCGHAZ is less than 100 J. As shown in Figure 5a–c, with increasing Cr content, fine-grained GB was observed in both 0Cr steel and 0.1Cr steel, whereas in 0.4Cr steel, fine-grained GB was predominant, accompanied by a small amount of BF. The M-A constituent, as a typical brittle hard phase, is distributed at the grain boundaries in a variety of morphologies (Figure 5b–f). It is noteworthy that the volume fraction of the M-A constituent gradually increases with the increase of Cr content, and the degree of aggregation becomes more significant. In this case, the presence of the M-A constituent tends to disrupt the continuity of the matrix and induces different degrees of lattice distortion in the matrix around the second phase. Such distortions will inevitably have an impact on the fracture behavior of the material, thus weakening the toughness of the material.

**Figure 6:**
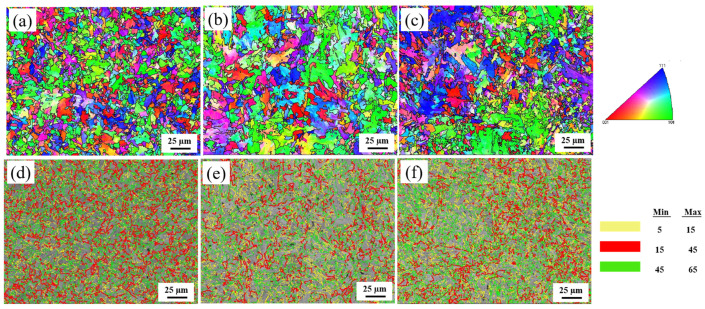
EBSD analysis of X80 experimental steels with different Cr contents in the SCCGHAZ (1000 °C): (**a**,**d**) 0Cr steel; (**b**,**e**) 0.1Cr steel; (**c**,**f**) 0.4Cr steel.

**Figure 7:**
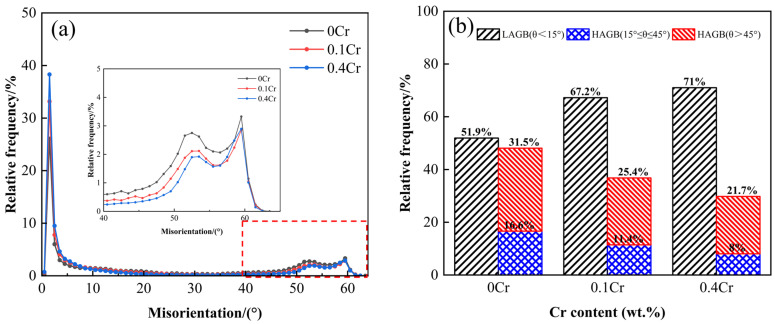
Grain boundary characteristics of X80 experimental steels with different Cr contents in the SCCGHAZ (1000 °C): (**a**) grain boundary misorientation angles; (**b**) grain boundary percentage.

To further analyze the crystallographic features and grain boundary properties, an EBSD analysis was performed. Figure 6a–c show the inverse polarity figures (IPF) of the three experimental steels at a peak temperature of 1000 °C. It can be seen from the figure that the misorientation difference between the two neighboring grains of 0Cr steel is larger and more uniformly distributed. In contrast, 0.1Cr steel has an increased number of grains on the (101) plane, while the grains of 0.4Cr steel are mainly dominated by the (111) and (101) planes, and the difference in grain misorientation between neighboring grains is smaller. Figure 6d–f show the grain boundary distribution, with the LAGB indicated by yellow (5–15°) lines and the HAGB indicated by red (15–45°) and green (45° < θ) lines. The grain boundary maps show that the grain boundary of GB exhibits an HAGB, in which 0Cr steel has the most uniform distribution of the LAGB and HAGB. The misorientation angle distribution curve and proportion statistics for the LAGB and HAGB are displayed in Figure 7. As described in Ref. [32], HAGBs with misorientation angles greater than 45 degrees can effectively retard or deflect crack propagation. Therefore, a misorientation angle of 45° is taken as the basis, as shown in Figure 7b. When Cr content is 0 wt.%, the HAGB proportion is highest (θ > 45°, 31.5%), and when the Cr content is 0.4 wt.%, the HAGB proportion is lowest (θ > 45°, 21.7%).

As an important organizational unit in pipeline steel, EGS plays a positive role in material toughness. According to the Hall–Petch formula (Equation (1)), it can be seen that the fine grain size is conducive to the improvement of strength but can also effectively inhibit crack extension to obtain more excellent comprehensive mechanical properties [33].

(1) $$\sigma_{c}=\sigma_{0c}+k_{c}d^{-1/2}$$

**Figure 8:**
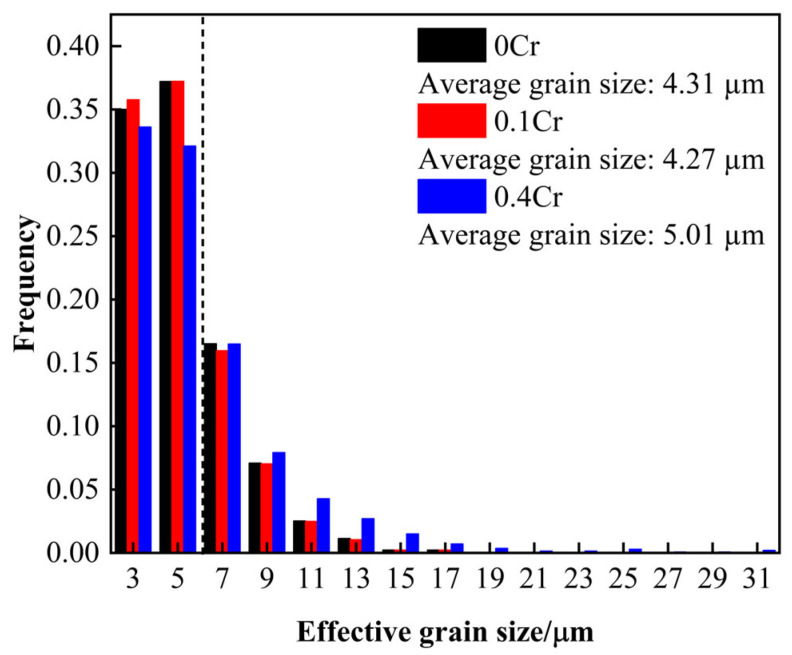
Distribution of effective grain sizes for X80 experimental steels with different Cr contents in the SCCGHAZ (1000 °C).

Here, *σ* is the cracking extension energy, d is the EGS, and $k_{c}$ is a material constant related to crystal type. The EGS is closely related to the density of the HAGB. A higher proportion of HAGBs leads to a finer EGS, thereby increasing the grain boundary length per unit area and elevating grain boundary energy. This enhances the cleavage strength and significantly improves the low-temperature toughness of the material [34,35]. HKL-Channel5 software is used to export EGS data, and the statistical results are shown in Figure 8. It can be seen that the average EGS increases with the increase of Cr content. When the Cr content is 0.4 wt.%, the average EGS is larger, at 5.01 µm, which may be due to the promotion of bainite growth by high Cr content. In addition, when the Cr content is 0 wt.% and 0.1 wt.%, the proportion of grains with an EGS of ≥7 µm is 27.78% and 27.01%, respectively; when the Cr content is 0.4 wt.%, the proportion of grains with an EGS of ≥7 µm is 34.26%. According to Equation (1), the smaller the EGS, the stronger the inhibition effect on cleavage crack propagation. However, the bainite grain size of 0.4Cr steel is higher than that of 0Cr steel and 0.1Cr steel, which not only reduces the number of grain boundaries per unit volume but also reduces the interfacial resistance to be overcome for crack propagation, resulting in the impact toughness of 0.4Cr steel being lower than that of 0Cr steel and 0.1Cr steel.

**Figure 9:**
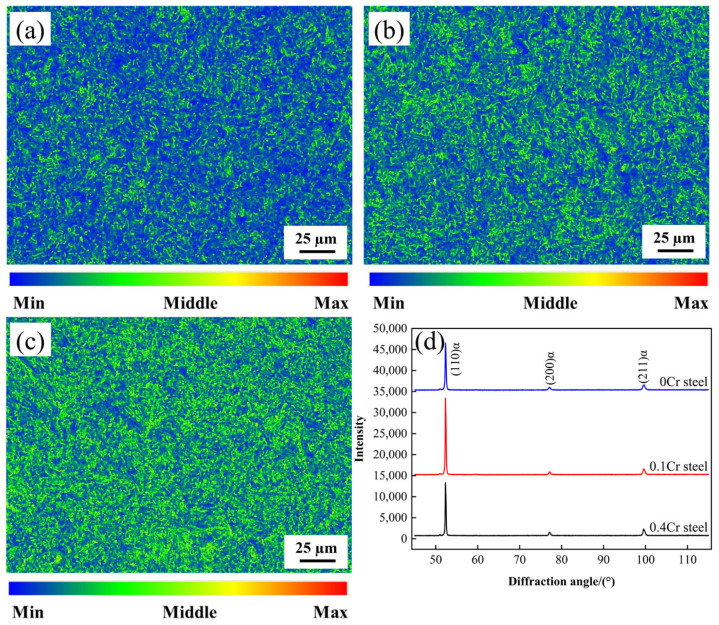
KAM maps of (**a**) 0Cr steel; (**b**) 0.1Cr steel; (**c**) 0.4Cr steel; and (**d**) XRD patterns.

The Kernel Average Misorientation (KAM) map obtained through EBSD analysis can qualitatively reflect the degree of lattice distortion and the distribution of dislocation density. Generally speaking, a higher KAM value indicates more significant local orientation gradients, which typically correspond to greater dislocation densities. KAM maps (Figure 9a–c) demonstrate that with increasing Cr content, the area fraction of light yellow regions increases while blue regions decrease, indicating elevated dislocation density. Notably, the dislocation density shows a positive correlation with microhardness (Figure 4b). KAM maps (Figure 9a–c) demonstrate that with increasing Cr content, the area fraction of light yellow regions increases while blue regions decrease, indicating elevated dislocation density. Notably, the dislocation density shows a positive correlation with microhardness (Figure 4b). This is primarily attributed to Cr promoting the formation of M-A constituents. When the volume fraction of M-A constituents is high, the martensite phase within these M-A constituents enhances the matrix hardness due to its high inherent hardness and elevated dislocation density. To quantitatively analyze the dislocation density, ρ, in the sample, XRD technology was used for analysis in this experiment. Figure 9d presents the XRD patterns of 0Cr steel, 0.1Cr steel, and 0.4Cr steel, where (110), (200), and (211) are the three strongest diffraction peaks of the bcc phase. The improved Williamson Hall Equation [36] can be used to calculate ρ (Equation (2)).

(2) $$\Delta K≅\frac{0.9}{D}+Nb\sqrt{\rho }K$$

Here, ΔK = 2cos θ (Δd)/λ, K = 2sin θ/λ, Δd is the full width at half maximum (minus instrument broadening), θ is the diffraction angle, λ is the wavelength of the incident light, D is the average grain size, N is a constant (0.263), and b is the Burgers vector. According to the calculation, the dislocation density of 0Cr steel is 6.2052 × 10^9^ cm^−2^, the dislocation density of 0.1Cr steel is 1.9075 × 10^10^ cm^−2^, and the dislocation density of 0.4Cr steel is 6.4796 × 10^10^ cm^−2^.

### 3.3. Fracture Analysis

**Figure 10:**
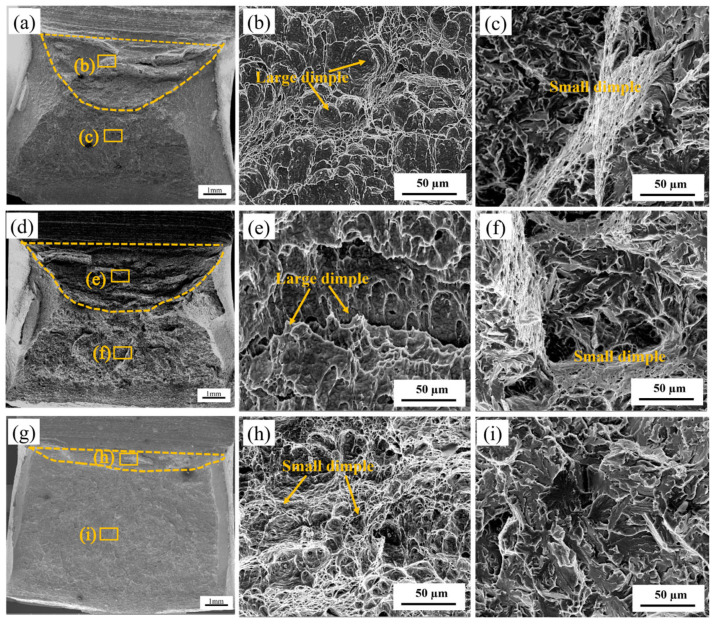
Impact fracture morphology of X80 experimental steels with different Cr contents in the SCCGHAZ (1000 °C): (**a**–**c**) 0Cr steel; (**d**–**f**) 0.1Cr steel; (**g**–**i**) 0.4Cr steel.

To conduct a comprehensive observation of the fracture morphology and fracture mode, SEM images of three experimental steels at a peak temperature of 1000 °C are shown in Figure 10. The fracture surface includes a fibrous zone, a radial zone, and a shear lip zone [37]. The fibrous zone involves a normal fracture under plane strain, usually in the shape of a “heel” with a deep center and narrow sides; the radial zone follows closely below the fibrous zone, where crack propagation changes from slow to rapid unstable propagation; and the shear lip zone involves shear fracture under plane stress, presenting a smooth shear surface, which is 45° to tensile stress. The larger the fiber zone and shear lip zone, the greater the toughness of the material. The impact test data shows that the average impact energy of 0Cr steel and 0.1Cr steel is 224.0 J and 203.9 J, respectively, exhibiting typical ductile fracture characteristics (Figure 10a,d). The average impact energy of 0.4Cr steel is 52.2 J, and the fracture surface is relatively flat, mainly composed of a fiber zone and radial zone, with almost no shear lip zone (Figure 10g). Therefore, 0.4Cr steel has the lowest toughness. Further observation reveals that the fibrous zone (Figure 10b,e,h) is composed of large and small dimples. The large and deep dimples indicate that the material can absorb more energy during fracture. However, with an increase in Cr content, the number of large dimples decreases, particularly when the Cr content is 0.4 wt.%. The radial zone (Figure 10c,f,i) exhibits quasi-cleavage fracture characteristics. The radial zone (Figure 10c,f,i) presents a quasi-cleavage fracture characteristic. In the 0Cr steel and 0.1Cr steel, continuous dimple bands in the radial zone indicate strong crack propagation resistance. However, in the 0.4Cr steel, the radial zone, which is mainly composed of cleavage facets and a few tear ridges, shows weak crack propagation resistance.

## 4. Discussions

### 4.1. Evolution of Microstructural Features During the Thermal Simulation Process

For the X80 experimental steel studied, its initial microstructure is mainly composed of small BF, GB, and M-A constituents, which provide good strength and toughness to the matrix. However, after undergoing a secondary thermal cycle with a peak temperature of 1000 °C, 0Cr steel and 0.1Cr steel formed GB with smaller grain sizes, while 0.4Cr steel had a higher Cr content, which promoted the formation of BF and resulted in GB with smaller grain sizes as the main component, while generating a small amount of BF [38]. The microscopic characterization results showed that with the increase in Cr content, the HAGB density of GB decreased, while the volume fractions of the EGS and M-A constituents increased. Among them, the EGS of 0Cr steel and 0.1Cr steel is relatively small, and there is a high-density HAGB. The combined effect of these two factors not only increases the effective area of grain boundaries that hinder crack propagation but also significantly enhances the ability to hinder cleavage crack propagation. Meanwhile, M-A constituents are mainly island-shaped, effectively improving the impact toughness of metals. Therefore, the fracture surface on the hammering side at the root of the V-shaped notch exhibits ductile fracture (Figure 10a,d). Under the same conditions, the HAGB density of 0.4Cr steel is lower, resulting in a larger EGS, while the volume fraction of the M-A constituent is too high, leading to a decrease in the toughness of the material. Therefore, the fracture surface on the hammering side at the root of the V-shaped notch exhibits quasi-cleavage fractures (Figure 10c).

### 4.2. Effect of M-A Constituents on Impact Toughness

**Figure 11:**
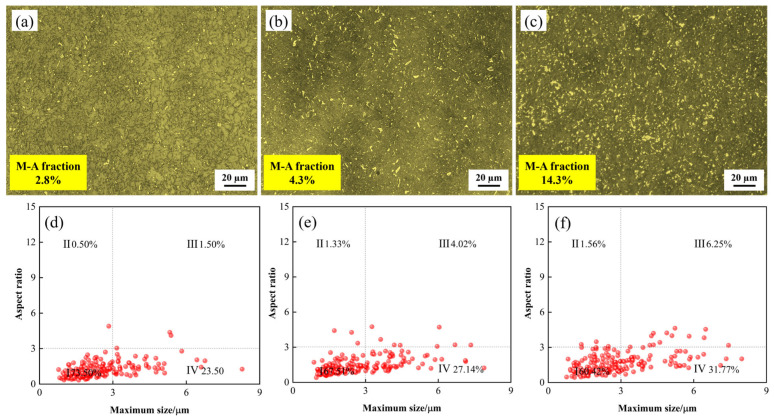
Distribution and morphology statistics of M-A constituents in the SCCGHAZ (1000 °C) of X80 experimental steels with different Cr contents: (**a**,**d**) 0Cr steel; (**b**,**e**) 0.1Cr steel; (**c**,**f**) 0.4Cr steel.

As shown in Figure 4, the matrix microstructure of the three experimental steels in the SCCGHAZ is predominantly GB. The main differences lie in the morphology, volume fraction, and size of the M-A constituents. After etching with LePera reagent, the micro-morphology of the M-A constituent is revealed. From Figure 11a–c, it can be seen that the M-A constituent is mainly concentrated in the grain boundary region and uniformly distributed in a dispersed state. In addition, with the increase in Cr content, the volume fractions of the M-A constituents are 2.8%, 4.3%, and 14.3%, respectively. The content of M-A constituent in 0.4Cr steel is significantly higher than that in 0Cr steel and 0.1Cr steel, resulting in relatively low impact energy of 0.4Cr steel. To characterize the morphology of the M-A constituent, following reference [39], they were classified into island, finestringer, coarse-stringer, and massive types. Obviously, the M-A constituents in the three experimental steels are mainly in island and block shapes, with almost no elongated M-A constituents. Refs. [40,41] suggests that the island-shaped M-A constituent can suppress crack propagation and thus have a positive effect on material toughness. In contrast, the blocky M-A constituent is prone to stress concentration at the interface with the matrix, leading to cracking, which is detrimental to the toughness of the material [42,43]. With increasing Cr content, the proportion of island-shaped M-A constituents decreased significantly, while that of blocky M-A constituents increased, which was accompanied by a reduction in impact energy. In summary, this study suggests that island-shaped M-A constituents are beneficial for improving toughness by inhibiting crack propagation, whereas blocky M-A constituents are detrimental to ductility and toughness.

**Figure 12:**
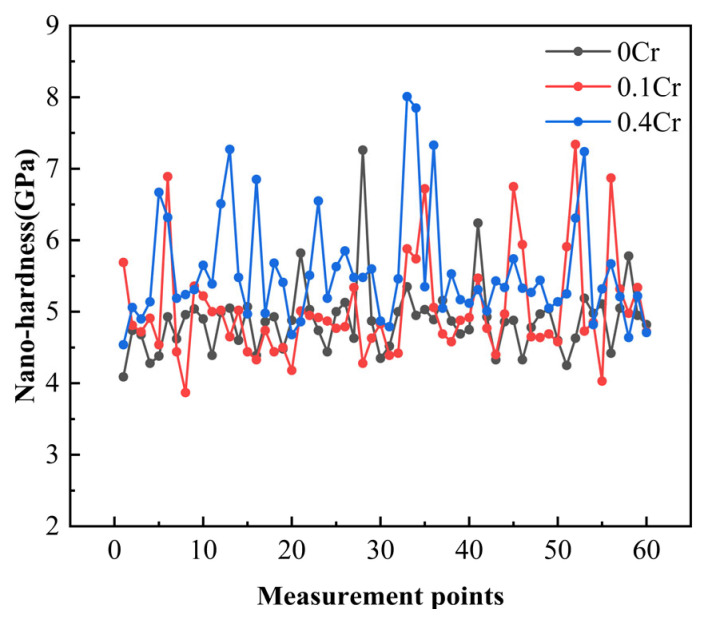
Comparison of nanohardness experimental steels with different Cr contents in the SCCGHAZ (1000 °C).

**Figure 13:**
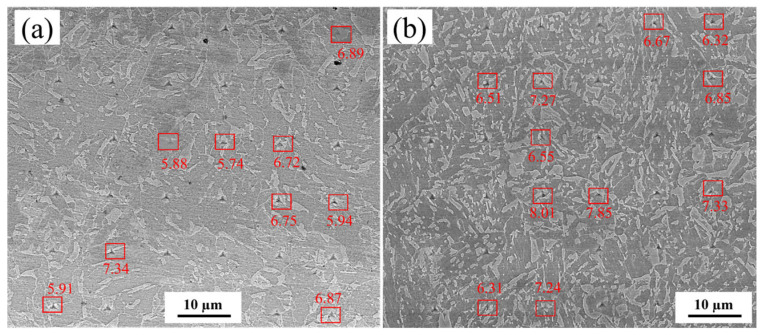
Nanoindentation locations for (**a**) 0.1Cr steel and (**b**) 0.4Cr steel.

Figure 12 presents the nanohardness distribution maps for the three experimental steels. Overall, the nanohardness of 0Cr steel and 0.1Cr steel is lower than that of 0.4Cr steel, similar to the change in microhardness. However, there are significant fluctuations in nanohardness at different positions, which may be due to the dependence of hardness on the inherent characteristics of each grain, including the non-uniformity of crystal orientation and dislocation density [44]. The nanoindentation hardness in some areas of Figure 12 is relatively high, which may be caused by the M-A constituent. The in situ nanoindentation results obtained via high-resolution SEM for 0.1Cr steel and 0.4Cr steel are presented in Figure 13, where indentations on M-A constituents are marked with red rectangles. It can be clearly observed that the nanohardness range of M-A constituents in 0.1Cr steel is 5.74–6.74 GPa, while the microhardness of M-A constituents in 0.4Cr steel is generally higher, maintaining a range of 6.31–8.01 GPa. When subjected to external forces, the interface between the harder M-A constituents and the softer matrix tends to develop significant stress. This can cause the M-A constituent to de-adhere from the matrix. The de-adhesion sites may form pits, which can trigger brittle fractures.

## 5. Conclusions

(1) In the SCCGHAZ, recrystallization and microstructure homogenization are promoted. Therefore, the peak temperature of the secondary thermal cycle has little effect on microhardness, and the hardness value remains at a relatively stable level. With the increase in Cr content, the dislocation density and volume fraction of M-A components increase, which makes the microhardness of experimental steel increase correspondingly. At the same time, the impact energy increases first and then decreases with the increase in the peak temperature of the secondary thermal cycle, and then it decreases gradually with the increase in the Cr content.
(2) After two recrystallizations, fine grains mainly composed of GB were formed in the SCCGHAZ. With the increase in Cr content, the proportion of the HAGB decreased, and the average EGS increased; specifically, when the Cr content was 0.4 wt.%, the HAGB content decreased to 29.7%, and the average EGS increased to 5.01 µm.
(3) An increase in the volume fraction of M-A constituents, a higher proportion of blocky M-A constituents, and an elevation in their nanohardness led to a reduction in impact energy. When the Cr content reached 0.4 wt.%, the impact energy decreased to below 100 J.

## Figures and Tables

**Table 1:** Chemical composition of the test steel with different Cr content (wt.%).

Pipeline No.	C	Si	Mn	Cr	Ni	Mo	Nb	Ti	Ceq
0Cr	0.060	0.23	1.67	0.01	0.21	0.20	0.073	0.015	0.370
0.1C	0.065	0.23	1.70	0.13	0.22	0.20	0.078	0.014	0.390
0.4Cr	0.060	0.21	1.73	0.40	0.22	0.21	0.074	0.013	0.456

## Data Availability

The original contributions presented in the study are included in the article, further inquiries can be directed to the corresponding authors.
